# Characterising Long-Term Depressive Symptoms Post-brain Injury: A Systematic Review of Symptom Trajectory Groups and Their Predictors

**DOI:** 10.1007/s11065-025-09674-6

**Published:** 2025-08-05

**Authors:** Priscilla Prince, Kristin Naragon-Gainey, Rodrigo Becerra, Michael Weinborn, Carmela F. Pestell

**Affiliations:** School of Psychological Science, The University of Western Australia, Perth, Australia

**Keywords:** Acquired brain injury, Depressive symptoms trajectories, Longitudinal, Predictors, Systematic review

## Abstract

This systematic review investigates the long-term trajectories of depressive symptoms in individuals with acquired brain injury (ABI) and identifies factors predicting group membership in these trajectories. The review follows the PRISMA guidelines and is registered on the International Platform of Registered Systematic Review and Meta-analysis Protocols (INPLASY-2023–11-0013). A comprehensive search of MEDLINE, PSYCINFO, EMBASE, CINHALPlus, ScienceDirect, Scopus, and Web of Science identified peer-reviewed studies published in English on adults aged 16 and above with an ABI diagnosis. Studies were included if they used a validated depression measure, had at least three assessment points, and applied group-based trajectory modelling. Exclusion criteria included studies focusing on neurodegenerative or neurodevelopmental disorders, or solely on treatments. The methodological quality was assessed using Joanna Briggs’ critical appraisal tool. The review synthesised data from ten studies involving 13,205 participants (average age 51.38 years, 55.86% male). Four depressive symptom trajectory groups were identified with varying prevalence: *stable low* (68%), *persistent high* (13%), *increasing* (20%), and *decreasing* (11%). Several key predictors including sex, age, injury severity, and education emerged as significant predictors of group membership in the *persistent high*, *increasing*, and *decreasing* depressive groups. However, variability in study methodologies and sample compositions posed challenges to direct comparison. Nonetheless, the review underscores the importance of long-term monitoring and the development of tailored interventions, as depression can manifest or intensify years post-injury. Understanding depressive symptom trajectories could help create personalised interventions, improving quality of life for those with depression after ABI.

**Supplementary Information:**

The online version contains supplementary material available at https://doi.org/10.1007/s11065-025-09674-6.

## Introduction

Acquired brain injury (ABI) is one of the leading causes of disability worldwide (Australian Institute of Health & Welfare, 2004). ABI refers to damage to the brain after birth, excluding congenital and degenerative conditions (Rees et al., 2007). The prevalence of ABI is steadily growing (Andelic et al., 2018), with traumatic brain injuries and stroke being common causes (Australian Institute of Health & Welfare, 2004). Traumatic brain injury (TBI) refers to damage to the brain resulting from an external force to the head (Guan et al., 2023), while stroke entails damage to the brain due to an interruption or reduction of blood supply to the brain (Li et al., 2022). Globally, there are 27 million new incidences of TBI (Guan et al., 2023) and 12 million new cases of stroke each year (Global Burden of Disease Collaborative Network, 2019). Other less common causes of ABI include hypoxia, tumours, and alcohol or drug poisoning (Watson et al., 2020). In Australia, TBIs alone have an approximate lifetime cost between $2.5 million to $4.8 million per injured individual (Department of Health & Aged Care, 2019). Most ABIs are heterogeneous, with mechanisms of focal and/or diffuse injuries occurring simultaneously (McKee & Daneshvar, 2015; Skandsen et al., 2010). As a result, disruption in communication both between and within brain regions is widespread, leading to a cascade of sequelae including cognitive deficits, movement impairments, and psychiatric conditions (Andelic et al., 2016).

Depression is one such highly prevalent sequelae in adults with ABI across all severity levels, affecting more than half of this population with such symptoms (Musliner et al., 2016; Scholten et al., 2016). These depressive symptoms, in turn, contribute to long-term functional impairments, hindering the individual’s quality of life and capacity to return to their functioning before injury, especially in more moderate to severe brain injuries (Juengst et al., 2015; Ponsford et al., 2014). Depression is a mood disorder characterised by persistent feelings of sadness, hopelessness, and loss of interest in previously enjoyed activities (American Psychiatric Association, 2013). Moreover, depression can lead to cognitive deficits including impairments in memory, attention, processing speed, and decision making (Lam et al., 2014). Depression manifests as a multidimensional construct through either neuropathological causes, situational/reactive factors, or a combination of both after a brain injury (McKee & Daneshvar, 2015; Roy et al., 2018).

Several neuropathological mechanisms of depression in adults after an ABI have been studied. For example, neuroendocrinal and chemical changes, including serotonergic and cholinergic deficits, occur during the acute period following a brain injury which can potentially contribute to depressive symptoms (Jorge & Starkstein, 2005). Additionally, injuries to the frontotemporal cortex, limbic system, and hippocampus, with corresponding widespread axonal damage have been significantly associated with depression. This is likely attributable to the crucial roles these brain regions play in regulating emotions (Griffis et al., 2019; Smith et al., 2019; Tham et al., 2011). Moreover, impairments in functional connectivity between brain networks including the frontoparietal network and default mode network have been correlated with depressive symptoms after a brain injury (Anand et al., 2005; Kaiser et al., 2015).

Furthermore, situational or reactive mechanisms can increase depressive symptoms following a brain injury (Bombardier et al., 2010). Functional impairments following ABI may limit the individual’s ability to engage in previously rewarding activities including occupational roles (Kreutzer et al., 2003) and socialising (Winkler et al., 2005). As a result, affected individuals may experience social isolation and financial strain, which may perpetuate their depressive symptoms (Ponsford et al., 2014). These limitations also commonly lead to decreased quality of life (Bombardier et al., 2010; Juengst et al., 2015), disability (Andelic et al., 2018), and increased dependence on others for activities of daily living (Ponsford et al., 2014). Furthermore, individuals after a brain injury often resort to various maladaptive coping strategies including isolation, denial, and emotional suppression when faced with new or stressful situations. Unfortunately, these coping mechanisms may inadvertently compromise their ability to effectively regulate negative emotions or stress (van der Horn et al., 2016), which may perpetuate depressive symptoms (Sasse et al., 2014).

The prevalence of depression in adults within the first year after a brain injury varies significantly in the literature, with reported rates ranging from 15% (Rapoport et al., 2003a, 2003b) to 53% (Bombardier et al., 2010). A systematic review conducted by Scholten et al. (2016) revealed that 17% of individuals met the criteria for depression in the first year after a TBI, and this rate escalated to 43% over time, highlighting the consistent and elevated rate of depression in individuals affected by a brain injury.

Depression is a dynamic mental health disorder characterised by changes in severity, duration, and symptoms over time (West et al., 2010). Longitudinal studies at a group level investigating individuals following a brain injury have produced diverse findings. Some have reported an increase in depressive symptoms (Gould et al., 2011a, b), while others have shown stability (Hart et al., 2012), and some have revealed improvements in symptoms (Rapoport, 2012). Moreover, risk factors have been identified that are associated with persistent depressive symptoms in adults after a brain injury including sex (Albrecht et al., 2019; Scholten et al., 2016), age (Scholten et al., 2016), and premorbid psychiatric history (Bombardier et al., 2010; Rapoport, 2012). Yet, the existing body of research presents conflicting findings. For example, certain studies have found that men are at an elevated risk of depression after a brain injury (Albrecht et al., 2019), while others assert that females are more susceptible (Scholten et al., 2016). Moreover, some studies have revealed that adults under the age of 65 with a brain injury are more likely to experience depression (Rapoport et al., 2003a, b; Scholten et al., 2016), whereas other studies propose that individuals over the age of 65 experience greater depressive symptoms (Albrecht et al., 2019). These study differences could be due to variability in methodology and sample composition. Nonetheless, these various outcomes underscore the intrinsic complexity and heterogeneity in how individuals may experience and cope with depressive symptoms after a brain injury.

A plausible reason accounting for these diverse findings is that previous studies assume that the entire population follows the same depression trajectory, with various predictors affecting each individual in the same way (Nagin & Odgers, 2010; Ren et al., 2017). This approach does not account for the heterogenous nature of brain injuries and so, the results may provide an inaccurate estimate of change in the symptoms of depression over time (Ren et al., 2017). To account for heterogeneity within the ABI population, some studies have employed group-based trajectory modelling, a statistical technique that assumes that a population contains distinct groups of individuals who follow similar progressions (within groups) over time (Nagin & Odgers, 2010; Ren et al., 2017). Group-based trajectory modelling is an analytical approach designed to identify latent classes of individuals who follow specific trajectories over time within a given sample (Nagin & Odgers, 2010). This model does not assume that every individual in a population follows the same trajectory; rather, it assumes that the sample is composed of homogenous subgroups, each demonstrating unique trajectory patterns (Nagin & Odgers, 2010). Group-based trajectory modelling includes latent class growth analysis and growth mixture models (Jung & Wickrama, 2008)*.*

**Fig. 1:**
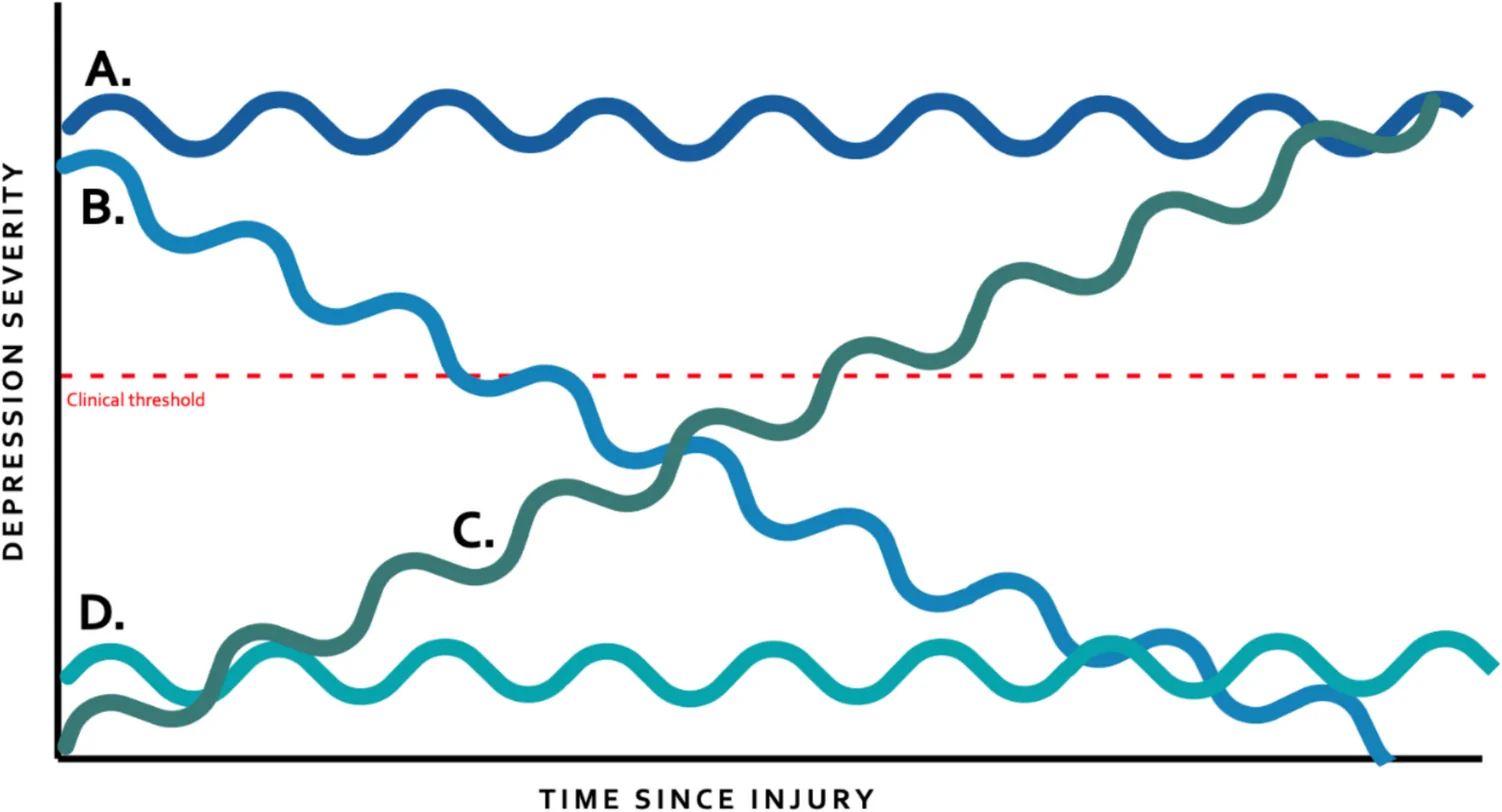
Visual depiction of various possible depressive symptoms trajectory group after an ABI. *Note.* Each line represents a distinct group of adults who follow a particular depressive symptoms trajectory after a brain injury. This figure represents some but not all classes that have been identified in the literature. These classes include individuals with: A, persistently high depressive symptoms; B, improving depressive symptoms; C, worsening symptoms; and D, stable low depressive symptoms. The red line represents the diagnostic clinical threshold for clinical depression

The existing body of research on depressive symptom trajectories within the ABI population has identified various classes including the following: stable low depressive symptoms (Ayis et al., 2019; Bombardier et al., 2016; Gomez et al., 2017; Kumar et al., 2021; Ren et al., 2017), persistently high depressive symptoms (Ayis et al., 2019; Gomez et al., 2017; Li et al., 2022; Ren et al., 2017) and improving symptoms (Bombardier et al., 2016; Kumar et al., 2021; Li et al., 2022) (see Fig. 1). However, some studies have also reported additional classes characterised by delayed onset of depression (Bombardier et al., 2016; Gomez et al., 2017) while others have revealed a class with rapidly worsening depression (Heath et al., 2023). Notably, groups characterised by delayed and high depression exhibit substantial fluctuations in depression levels over time, occasionally falling below clinically significant thresholds (Gomez et al., 2017). However, the number of depressive symptom trajectory groups varies across studies, ranging from two (Shen & Wang, 2023) to five (Li et al., 2022) depressive groups. Additionally, the prevalence of these depressive groups varies substantially between studies. For example, some studies have found that over 70% of participants belong to a low depressive symptom trajectory group, suggesting that a majority experience minimal depressive symptoms over time (Gomez et al., 2017; Heath et al., 2023). In contrast, another study has revealed that 29% of their sample belonged to the same group, indicating a much lower prevalence of minimal depressive symptoms and suggesting a higher burden of depressive symptoms among these individuals (Ayis et al., 2019). To improve our understanding of the natural course of depressive symptoms after a brain injury, it is crucial to systematically characterise and consolidate these groups.

Investigating specific depressive trajectory classes can aid in identifying specific predictors that may perpetuate or reduce depressive symptoms over time (Gomez et al., 2017). For instance, research has shown that women with brain injuries tend to have more significant and persistent depression (Gomez et al., 2017), whereas men with brain injuries show increasing depressive symptoms over time (Heath et al., 2023). Additionally, middle-aged individuals (between 40 and 60 years old) are more likely to belong to the persistent depression group which is possibly attributed to significant disruptions in daily life roles such as occupational, parenting, and community engagement challenges (Bombardier et al., 2016; Carmichael et al., 2023). Furthermore, the persistent and delayed depression classes were significantly more likely to have a history of premorbid psychiatric disorders on the basis of antidepressant use and engagement in counselling (Bombardier et al., 2016; Carmichael et al., 2023). Despite these findings, evidence remains mixed regarding specific predictors such as sex and injury severity across depressive symptom trajectory groups (Bombardier et al., 2016; Gomez et al., 2017; Shen & Wang, 2023).

To effectively address and alleviate depressive symptoms in individuals living with a brain injury, it is imperative to enhance our understanding of the natural progression of depression following such injuries and the associated predictors (Bombardier et al., 2016; Ren et al., 2017). Employing a longitudinal study design allows for a comprehensive examination of how depression unfolds and changes in the aftermath of a brain injury (Ren et al., 2017). While individual studies provide valuable insights, they also present varying methodologies, sample sizes, and findings. This variability can make it challenging for readers to integrate results and understand the broader patterns in the literature. Conducting a systematic review holds the potential to consolidate and synthesise these studies, hence providing a comprehensive overview of the underlying patterns and trends in long-term depression trajectory after a brain injury and the critical gaps in the literature (Scholten et al., 2016).

Given that no review to date has examined the long-term trajectory of depressive symptoms in the ABI population, this review not only enhances the evidence base but also helps identify areas where crucial information might be lacking, particularly regarding the long-term variability in depression prevalence. Furthermore, gathering and analysing data concerning predictors in the context of depressive symptom trajectories subsequent to a brain injury contributes to a more nuanced and comprehensive understanding of the intricate interplay between individual attributes and depressive outcomes (Scholten et al., 2016). This approach not only provides valuable insight into the evolving nature of depressive symptoms but also equips researchers and clinicians with the ability to pinpoint individuals who are more vulnerable to depressive symptoms based on the trajectory group the individual is associated with. This may enable the development of personalised treatment tailored to the unique characteristics of each group (Sigurdardottir et al., 2014).

The aim of this systematic review is to (1) examine longitudinal patterns of depressive symptoms following an ABI, by identifying and characterising distinct groups of individuals who follow various trajectories of depressive symptoms, and (2) to identify the predictors associated with specific groups of depressive symptoms trajectory such as sex, age, and psychiatric history. Establishing a more nuanced understanding of these trajectory groups is vital for informing targeted interventions and advancing our understanding of the dynamic nature of depressive symptoms after a brain injury. By implementing targeted interventions, proactive steps can be taken towards minimising the persistence of depression and mitigating its impact on the individual’s overall quality of life. Moreover, targeted interventions may aid in reducing the global economic burden of brain injury by improving productivity and lowering healthcare costs.

## Methods

This review systematically examined the selected studies to identify and examine the most common depressive symptom trajectory groups after an ABI, including the characteristics and prevalence of each trajectory group. Additionally, the predictors associated with membership to specific groups were examined. The current systematic review adhered to the Preferred Reporting Items for Systematic Reviews and Meta-analyses (PRISMA) guidelines (Moher et al., 2009). A review protocol was prospectively registered on the International Platform of Registered Systematic Review and Meta-analysis Protocols (INPLASY) under the following title “Characterising the Trajectory of Depression and Associated Risk Factors Following an ABI in Adults: A Systematic Review and Meta-Analysis” (https://inplasy.com/inplasy-2023-11-0013/).

## Literature Search

A comprehensive search was conducted to identify relevant literature which examined depressive symptom trajectories in the ABI population using group-based trajectory modelling. The literature search was undertaken using online databases including MEDLINE, PSYCINFO, EMBASE, CINHALPlus, ScienceDirect, Scopus, and Web of Science. Search terms were related to ABI, depressive symptom trajectory, and group-based trajectory modelling (see Table S1 for details). Hand searching also took place to identify any relevant literature. To gather all relevant articles, the initial search in the online databases was broad with no restrictions on publication date or status.

## Study Selection

**Fig. 2:**
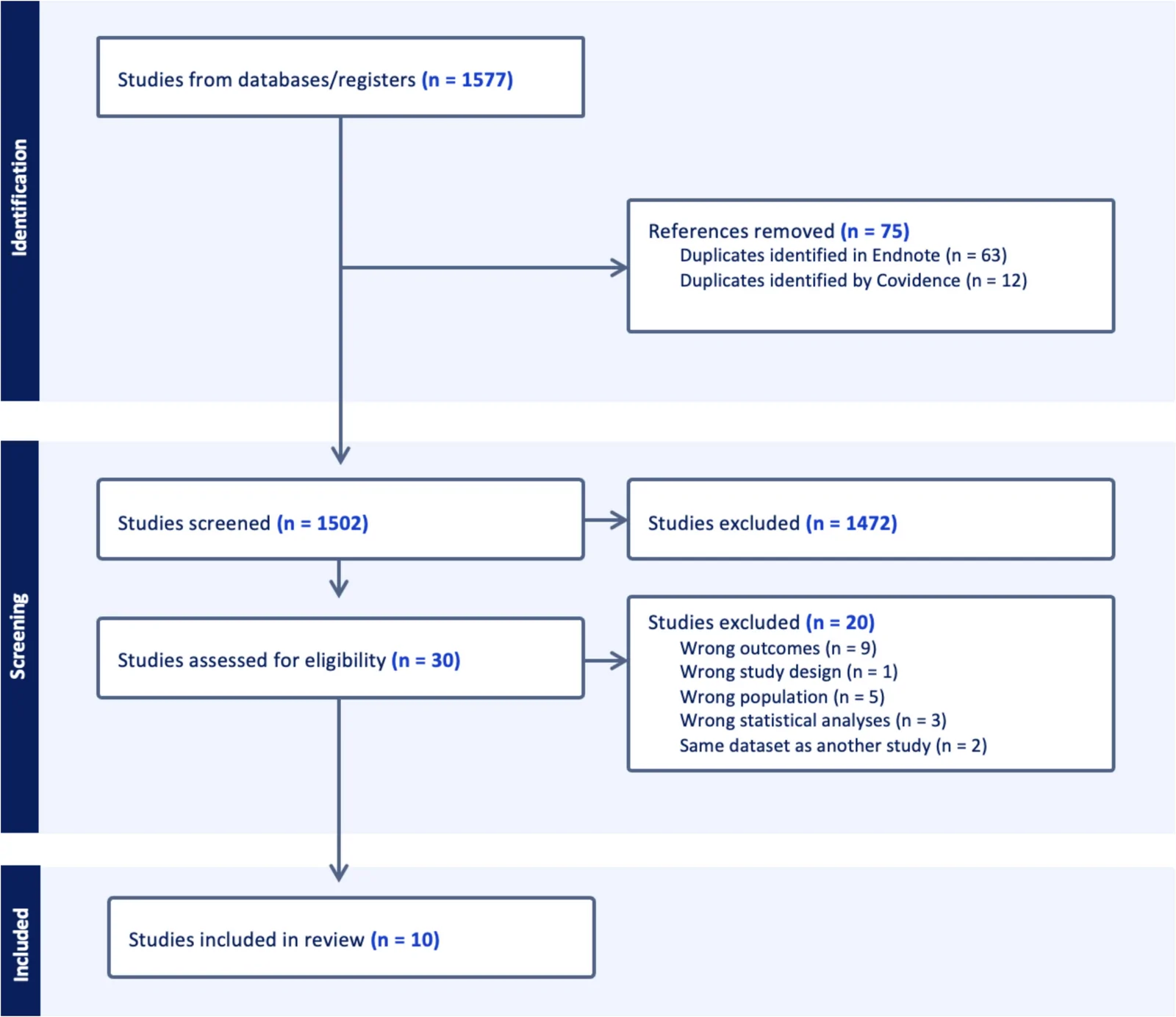
PRISMA study selection process

The study selection followed the PRISMA guidelines as depicted in Fig. 2. This included removing duplicate studies that were collected by the databases during the title screening process in EndNote. The search process was based on the following inclusion criteria to screen for abstracts and full-text studies: (a) peer-reviewed studies published in English with full text available; (b) an adult population (16 years old and above); (c) formal ABI diagnosis for participants; (d) a validated measure of depression, with depressive symptoms defined as any sub-clinical or clinical manifestations of depression, as evaluated through an established screening or diagnostic instrument based on the most recent version of the Diagnostic and Statistical Manual of Mental Disorders (DSM) (American Psychiatric Association, 2013) or the International Statistical Classification of Diseases and Related Health Problems (ICD) criteria (World Health Organisation, 2019); (e) the study has a minimum of three assessment time points; and (f) the study employed a group-based trajectory modelling analysis technique. Additionally, exclusion criteria included (a) participants with neurodegenerative conditions or neurodevelopmental disorders and (b) the study solely examined a treatment or intervention in the ABI population.

Covidence software was used to screen the studies (Covidence, 2023). The screening process for the selected studies was carried out collaboratively by two authors (PP and CP). All abstracts were initially screened by one author (PP), while both authors (PP and CP) independently reviewed all the full-text articles to ensure agreement was met on the inclusion criteria for the studies selected. Subsequently, data were extracted by one author (PP). Any discrepancies or uncertainties regarding the inclusion of studies or data extraction were resolved through discussions between the two authors (PP and CP) until a consensus was achieved. The search was conducted in November 2023, the screening and review processes were completed in December 2023, and data extraction was finalised in March 2024, and an additional search was completed in September 2024.

## Assessment of Methodological Quality

To evaluate the methodological quality of each study, predefined quality criteria outlined in Joanna Brigg’s critical appraisal tool for prevalence studies were employed (see Table S2) (Munn et al., 2020). This tool contains nine questions regarding the quality of each study, and each item is appraised as either ‘Yes’, ‘No’, ‘Unclear’, or ‘Not Applicable’. Risk of bias was assessed based on the proportion of ‘Yes’ responses to each item. Low risk of bias was determined if the studies had 70% or more ‘Yes’ responses, moderate risk was 50% to 69% ‘Yes’ responses, and high risk of bias was determined for studies with 49% or fewer ‘Yes’ responses (Polmann et al., 2019). Quality assessment was conducted independently by both authors, and any discrepancies were resolved through discussion until a consensus was reached. No studies were excluded based solely on methodological quality.

## Data Extraction

All extracted data from the selected studies were recorded on Covidence. The information extracted was study characteristics (year of publication, country, recruitment setting and method, journal, funding sources, reported conflicts of interest), sample characteristics (sample size, age, sex composition, educational history, premorbid psychiatric history), brain injury characteristics (injury type, injury severity, and time since injury), statistical modelling technique, and depressive symptom characteristics (self-report measure used, number of assessment timepoints, average score at each timepoint, number of trajectory groups).

The depressive symptom trajectory groups were synthesised based on the severity of depressive symptoms and their progression over time. The severity and progression of depressive symptoms were determined for most studies by whether participants scored above or below the clinical threshold for depression, as measured by specific self-report instruments used in each study, and by assessing changes in depressive symptom scores over time.

Due to the variability in sample sizes and the prevalence of depressive symptom trajectory groups across studies, the prevalence for each group was recalculated to provide a more accurate overall average. The recalculated prevalence was derived by aggregating the total number of individuals across all studies who belong to a specific depressive symptom trajectory group, and then dividing this sum by the total number of participants in the studies that identified that group. This approach offers a more precise representation of the percentage of individuals within each depressive symptom trajectory group.

## Results

Following a comprehensive literature search, 1577 studies were identified, of which 75 were duplicates and subsequently removed. Of the remaining 1502 studies, screening of the titles and abstracts led to the exclusion of 1472 studies. The full texts of the remaining 30 studies were then reviewed, resulting in the exclusion of 20 studies. The studies were excluded for the following reasons: nine studies investigated the wrong outcome, five did not include the intended target population, three did not use group-based trajectory modelling, two studies used the same dataset as another study which was included in the review, and one did not employ a longitudinal study design (Fig. 2). The Cohen’s Kappa agreement rate between the authors (PP and CP) on the full-text screening was 0.80, indicating a substantial agreement between both authors (McHugh, 2012). Data extraction was conducted by one author (PP), with discussion with the co-author (CP) when required. Overall, the quality assessment demonstrated low risk of bias for most studies (Tables 1 and S3). Both reviewers assessed the quality of all studies. The Cohen’s kappa agreement rate between authors was 0.36, indicating moderate agreement between both authors. The initial average percentage of agreement between authors was 69% which improved to 100% following discussion.

**Table 1:** Characteristics of the ten reviewed studies

Study	Brain injury type	Sample size	Mean age (SD)	Sex (% male)	Brain injury severity measure	Depression measure (cut-off score)	Injury severity %			No. of assessment timepoints	Follow-up points	Number of depressive symptom groups	Overall quality assessment score
							Mild	Mod	Severe				
Ayis et al. (2019) (Female sample)	Stroke	1038	65.09 (13.33)	0%	GCS	HADS (> 8)	-	-	-	6	3 months, 1, 2, 3, 4, 5 years	4	Low risk
Ayis et al. (2019) (Male sample)	Stroke	1275	68.87 (15.66)	55.0%	GCS	HADS (> 8)	-	-	-	6	3 months, 1, 2, 3, 4, 5 years	3	Low risk
Kang (2021)	Stroke	766	77.35 (6.54)	44.0%	-	CESD	-	-	-	3	Pre stroke, stroke reported, post-stroke	4	Low risk
Malhotra et al. (2016)	Stroke	191	71.6 (12.2)	48.8%	-	CESD (> 16)	-	-	-	3	0–10 weeks, 11–22 weeks, 23–47 weeks	3	Low risk
Bombardier et al. (2016)	TBI	559	42.5 (17.9)	71.6%	GCS	PHQ-9	52.1%	22.9%	22.9%	9	1,2,3,4,5,6,8,10, 12 months	4	Low risk
Heath et al. (2023)	TBI	148	38.7 (16.36)	76.0%	GCS & PTA	BDI (> 16)	0%	1%	99%	4	2, 5, 12, 36 + months	4	Moderate risk
Gomez et al. (2017)	TBI	1074	35.96 (16.61)	64.8%	PTA	HADS (> 8)	71.7%	26.6%	1.7%	6	Baseline, 1,3,6,12,24 months	3	Low risk
Kumar et al. (2021)	TBI	3541	73.35 (5.75)	40.9%	LOC	CESD (> 10)	-	-	-	6	Baseline, 2,4,6,8,10 years	4	Low risk
Martinez et al. (2024)	TBI	2585	39.7 (16.65)	67.4%	GCS	PHQ-9	86.1%	13.9%		4	2 weeks, 3, 6, 12 months	3	Low risk
Ren et al. (2017)	TBI	129	33.3 (14.6)	76.7%	GCS	BSI-19 (T score > 63)	-	100%		4	3, 6, 12, 24 months	2	High risk
Shen and Wang (2023)	TBI	1989	18.79 (1.58)	69.3%	GCS	PHQ-9	13.65%	8.37%	77.98%	8	1,2,5,10,15,20,25,30 years	2	Low risk

Note. Risk of bias was assessed based on the proportion of ‘Yes’ responses to each item. Low risk of bias was determined if the studies had 70% or more ‘Yes’ responses, moderate risk was 50% to 69% ‘Yes’ responses, and high risk of bias was determined for studies with 49% or fewer ‘Yes’ responses (Polmann et al., 2019). One study was deemed as high risk due to insufficient information provided to answer the quality assessment questions. The reported mean age reflects participants’ age at baseline. Percentage of injury severity was left blank for the studies that did not explicitly state the injury severity in the sample

Abbreviations: *GCS*, Glasgow Coma Scale; *PTA*, post-traumatic amnesia; *LOC*, loss of consciousness; *HADS*, Hospital Anxiety and Depression Scale; *CESD*, Centre for Epidemiologic Studies Depression Scale; *PHQ-9*, Patient Health Questionnaire; *BDI*, Beck’s Depression Inventory; *BSI-19*, Brief Symptom Inventory

## General Characteristics of Included Studies

The ten included studies are recent longitudinal studies published between 2016 and 2024. Six studies were conducted in the USA, two in the UK, and one each in Australia, Canada, and Singapore. The review included seven TBI studies and three stroke studies; no studies addressed other ABI conditions (Table 1).

Participant recruitment varied: six studies recruited participants from larger parent studies focusing on TBI (Heath et al., 2023; Ren et al., 2017; Shen & Wang, 2023), stroke (Ayis et al., 2019), and older adult populations (Kang, 2021; Kumar et al., 2021), while four studies recruited participants from specific hospitals (Bombardier et al., 2016; Gomez et al., 2017; Kang, 2021; Martinez et al., 2024). The inclusion and/or exclusion criteria were not explicitly stated for four studies (Gomez et al., 2017; Kang, 2021; Malhotra et al., 2016; Ren et al., 2017). However, all studies included participants who had sustained a first-time TBI or stroke of varying severity and had received a formal ABI diagnosis.

Overall, the ten studies consisted of 13,205 participants, with sample sizes ranging from 129 to 3541 participants. The average age of the participants was 51.38 years, ranging from 18 to 77 years old. The biological sex composition was fairly even, with 55.86% of the sample being of male sex. Brain injury severity was described in varying terms from very mild to severe. Although the methods of measuring brain injury severity differed across studies, most studies utilised either the Glasgow Coma Scale, loss of consciousness, and/or post-traumatic amnesia as part of their assessment criteria (further details on brain injury characteristics are available in supplementary materials in Table S4). Of the ten studies reviewed, six reported specific injury severity data. The distribution of severity levels appears relatively balanced: three studies reported a higher proportion of mild injuries, while the other three studies had a larger percentage of moderate to severe injuries (see Table 1).

## Measurement of Depression

All studies employed a self-reported measure to assess depression severity, with seven studies providing information on the cut-off scores for clinical depression (Table 1). The depression measures included were the Centre for Epidemiological Studies Depression (CESD) (Radloff, 1977), Patient Health Questionnaire (PHQ-9) (Kroenke et al., 2001), Beck Depression Inventory (BDI) (Robert et al.,^1987^), Hospital Anxiety and Depression Scale (HADS) (Zigmond & Snaith, 1983), and Brief Symptom Inventory (BSI-19) (Derogatis, 1993). Additionally, one study included questions on whether participants have been diagnosed with depression and whether they used antidepressants and/or engaged in therapy (Bombardier et al., 2016). The duration of longitudinal depression severity assessments varied across studies, ranging from a few months after the injury to up to 30 years post-injury. The number of assessment time points also varied, with the most common being six (range = 3–9 assessment timepoints). A meta-analysis was not conducted due to difficulties in obtaining the depression data for all studies. Of the authors who could be contacted, two granted permission to access their data, while one indicated that the raw dataset could not be shared. As a result, there was insufficient data available to perform a meta-analysis.

## Analytical Strategies

As per the inclusion criteria, all studies used group-based trajectory analyses as their primary statistical analysis. Notably, Ayis et al. (2019) examined depression trajectory groups separately for males and females. To account for these sex differences, the present review reported these subsamples separately. No other study in this review utilised this approach to examine depression groupings. With regard to determining the optimal number of depression trajectory groups, all studies considered the Bayesian Information Criterion. Seven studies examined predictors of depressive symptom group membership and used multinomial logistic regression analysis (Bombardier et al., 2016; Gomez et al., 2017; Heath et al., 2023; Kumar et al., 2021; Martinez et al., 2024; Ren et al., 2017; Shen & Wang, 2023). In these analyses, the *stable low* depressive group was the reference group, meaning all other groups were compared against this baseline group to assess the relative differences in depression trajectories.

## Depressive Symptom Trajectory Groups, Prevalence and Predictors

The number of depressive groups varied across studies, ranging from two to four. Half of the datasets identified four distinct depressive symptom trajectory groups after an ABI. Across studies, common themes in depressive symptom trajectories emerged. This review synthesised these trajectories into four distinct categories based on the severity of depressive symptoms and their changes over time. The four depressive symptom trajectory groups include the following: ‘stable low’, ‘persistent high’, ‘decreasing’, and ‘increasing’. Seven studies in this review found predictors of depressive symptom trajectory group membership, all of which were TBI studies. The study by Malhotra et al. (2016) was the only stroke study that examined predictors and found no significant predictors for group membership. The studies collected information on predictors of group membership at baseline.

### Stable Low Depressive Symptoms

The *stable low* depressive symptom group consistently had average depressive symptom scores well below the clinical cut-off point for depression at all assessment points, with these scores remaining relatively stable over time. Eight of the ten studies mentioned this group, referring to it variously as ‘stable low’ (Ayis et al., 2019; Heath et al., 2023; Martinez et al., 2024; Shen & Wang, 2023), ‘stable minimal’ (Heath et al., 2023), ‘minimal’ (Kumar et al., 2021), ‘low’ (Bombardier et al., 2016; Gomez et al., 2017), ‘resilience’ (Kang, 2021), and ‘moderate’ (Ayis et al., 2019). Although Ayis et al. (2019) identified both a low and moderate depressive symptom group, the moderate group was included in the *stable low* group due to its average depressive score of six, which is below the clinical threshold of greater than eight. The prevalence of the *stable low* group ranged from 29 to 86%, with a readjusted prevalence of 68.40% (8813 individuals), making it the most prevalent group compared to the others (Table 2). Because this group served as the reference group for predictors, results are described below in reference to each of the other trajectory classes.

**Table 2:** Depressive symptom trajectory groups and their prevalence

Study	Subsample size (total sample size)	Prevalence	Original trajectory name	Description
*Stable low depressive symptom trajectory group*				
Ayis et al. (2019) (*female* sample)	302 (1038)	29.09%	Low	-
Ayis et al. (2019) (*female* sample)	517 (1038)	49.81%	Moderate	-
Ayis et al. (2019) (*male* sample)	537 (1275)	42.12%	Low and Stable	-
Bombardier et al. (2016)	392 (559)	70.12%	Low	Depression scores were in the nondepressed range throughout the follow-up period
Heath et al. (2023)	15 (148)	10.0%	Stable Low	-
Heath et al. (2023)	104 (148)	70.0%	Stable Minimal	-
Gomez et al. (2017)	818 (1074)	76.2%	Low	Depression scores were well below clinical cut-off threshold
Kang (2021)	516 (766)	67.4%	Resilience	Low depression score across three timepoints
Kumar et al. (2021)	1693 (3451)	49.05%	Minimal	-
Martinez et al. (2024)	2228 (2585)	86.2%	Stable Low	Relatively low symptoms at first assessment through to the last assessment
Shen and Wang (2023)	1691 (1989)	85.0%	Low-Stable	-
*Persistent high depressive symptom trajectory group*				
Ayis et al. (2019) (*female* sample)	169 (1038)	16.28%	Stable Severe	-
Ayis et al. (2019) (*female* sample)	50 (1038)	4.82%	Stable Very Severe	-
Ayis et al. (2019) (*male* sample)	145 (1275)	11.37%	Severe Persistent	-
Bombardier et al.(2016)	35 (559)	6.26%	Persistent	Average depression scores remained in the moderately severe depression range throughout follow-up period
Gomez et al. (2017)	161 (1074)	15.5%	High	Depression scores well above clinical threshold overtime
Kang (2021)	68 (766)	8.9%	Chronic	Pattern of high depression scores over time
Kumar et al. (2021)	447 (3451)	12.65%	Persistent	-
Ren et al. (2017)	38 (129)	29.6%	High-stable	Depression scores started high and remained high with no significant change over time
*Increasing depressive symptom trajectory group*				
Ayis et al. (2019) (*male* sample)	593 (1275)	46.51%	Moderate Increasing	-
Bombardier et al. (2016)	74 (559)	13.24%	Delayed	Depression scores increasing from the nondepressed range during months 1–5 to the depressed range in months 6–12
Heath et al. (2023)	18 (148)	12%	Delayed	Minimal depressive symptoms in the first 12 months post-injury, symptoms increased significantly by the fourth and final timepoint
Heath et al. (2023)	12 (148)	8%	Rapidly Worsening	Low level of symptoms at first timepoint that increased over time
Gomez et al. (2017)	89 (1074)	8.3%	Delayed	Score increase in the slope in the first 12 months, followed by a sharp decrement. Although the trajectory for this class was below the clinically relevant cut-off score level initially and at 24 months, it was above the clinically relevant cut-off score over most of the 24 months and can be considered a delayed depression class
Kang (2021)	94 (766)	12.3%	Emergent	Increasing depression scores and experienced progressively severe depression over time
Kumar et al. (2021)	864 (3451)	25.05%	Increasing	-
Malhotra et al. (2016)	82 (191)	43.1%	Low and increasing	Curvilinear with depressive symptoms being highest at first assessment timepoint
Malhotra et al. (2016)	18 (191)	9.3%	High and increasing	Depression scores started high and sloped up over time
Martinez et al. (2024)	230 (2585)	8.9%	Worsening	Relatively low symptoms at first assessment with an increasing course until 12 months
Ren et al. (2017)	91 (129)	70.4%	Low-Increasing	Scores started low and gradually increased over time
Shen and Wang (2023)	298 (1989)	15%	High-Increasing	Characterised by a high intercept mean and the depression severity increased over time with a statistically significant slope mean
*Decreasing depressive symptom trajectory group*				
Bombardier et al. (2016)	58 (559)	10.38%	Recovery	Depression scores began in the depressed range but declined overtime, and by months 10–12, average scores were well in the non-depressed range
Kang (2021)	87 (766)	11.3%	Recovery	Depression improved over time, as the depression scores decreased
Kumar et al. (2021)	457 (3451)	13.25%	Decreasing	-
Malhotra et al. (2016)	91 (191)	47.6%	Low and decreasing	Depression scores started just above the clinical threshold and sloped down over time
Martinez et al. (2024)	127 (2585)	4.9%	Improving	Relatively high symptoms at first assessment with a progressive decline to levels of the stable low group by 12 months

### Persistent High Depressive Symptoms

Participants in this group consistently had depressive symptom scores in the moderate to severe range at each assessment time point. The studies used various labels for this group, including ‘persistent’ (Bombardier et al., 2016; Kumar et al., 2021), ‘high’ (Gomez et al., 2017), ‘high-stable’ (Ren et al., 2017), ‘chronic’ (Kang, 2021), ‘severe persistent’ (Ayis et al., 2019), ‘stable severe’ (Ayis et al., 2019), and ‘stable very severe’ (Ayis et al., 2019). This group was included in six studies and had a lower prevalence compared to the *stable low* depressive symptom group, ranging from six per cent to 29%, with an overall adjusted prevalence of 13.43% (1113 individuals) (Table 2).

Several predictors were identified for individuals with *persistent high* depressive symptoms after ABI (Table 3). Compared to the *stable low* depressive symptom group, Gomez et al. (2017) found that individuals in the *persistent high* depressive group spent fewer days in hospital. This study also found that being female and older age were significant predictors (Gomez et al., 2017). In contrast, Kumar et al. (2021) revealed that men with TBIs with loss of consciousness had a higher chance of being in the *persistent high* group. Additionally, the use of antidepressants and/or counselling was linked to a higher likelihood of *persistent high* depressive symptoms (Bombardier et al., 2016). Higher injury severity was associated with an increased likelihood of belonging to the *persistent high* depressive symptom group (Gomez et al., 2017; Ren et al., 2017).

**Table 3:**
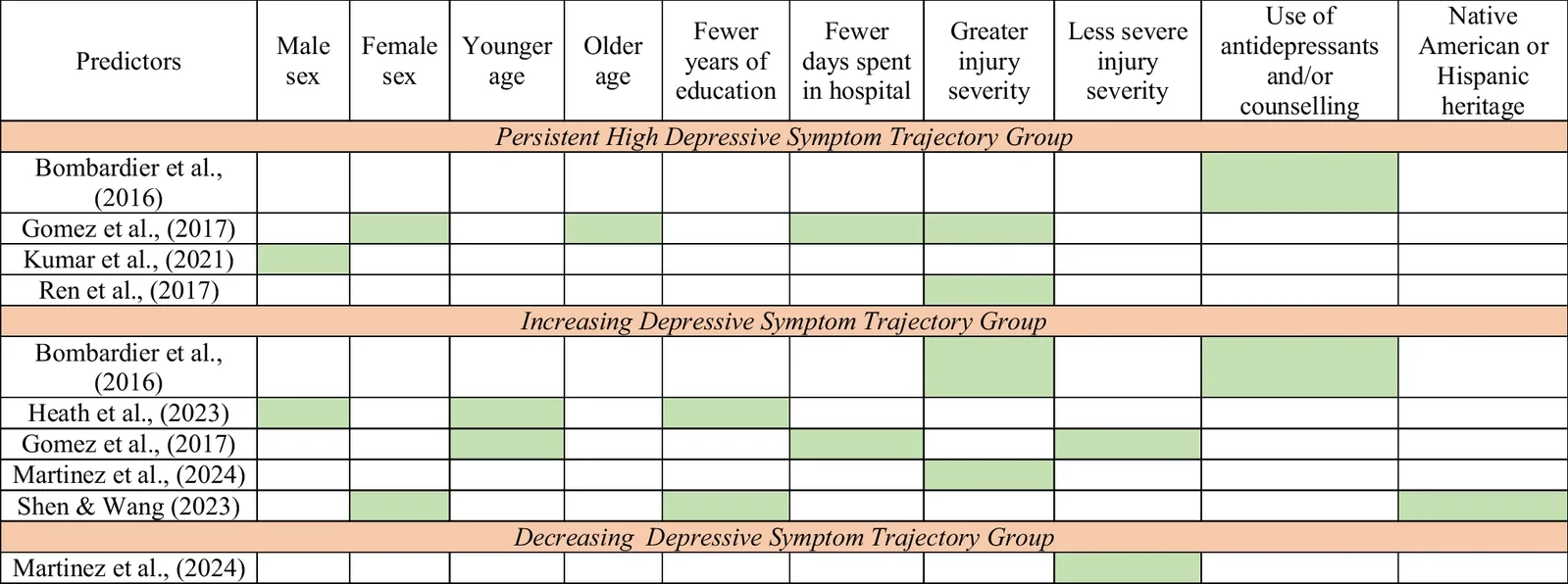
Predictors identified in the persistent high and increasing depressive symptom trajectory groups

### Decreasing Depressive Symptoms

The *decreasing* depressive symptom group initially had depression scores above the clinical threshold, which gradually declined below the threshold over time. Five studies included this group, labelling it as ‘decreasing’ (Kumar et al., 2021), ‘recovery’ (Bombardier et al., 2016; Kang, 2021), ‘improving’ (Martinez et al., 2024), and ‘low and decreasing’ (Malhotra et al., 2016). The prevalence of this group ranged from 5 to 47%, with an overall adjusted prevalence of 10.85% (819 individuals) (Table 2). Only one of the five studies in this group identified a predictor of group membership, which was having a less severe TBI (Martinez et al., 2024).

### Increasing Depressive Symptoms

All studies included a trajectory group with *increasing* depressive symptoms. In most cases, participants initially had low depressive symptom scores that gradually rose above the clinical threshold over time. However, two studies identified groups that had high initial scores that continued to increase in severity (Malhotra et al., 2016; Shen & Wang, 2023). Various names were used for this group including ‘high and increasing’ (Malhotra et al., 2016; Shen & Wang, 2023), ‘low and increasing’ (Malhotra et al., 2016; Ren et al., 2017), ‘worsening’ (Martinez et al., 2024), ‘moderate increasing’ (Ayis et al., 2019), ‘increasing’(Kumar et al., 2021), ‘rapidly worsening’(Heath et al., 2023), ‘delayed’ (Bombardier et al., 2016; Gomez et al., 2017; Heath et al., 2023), and ‘emergent’ (Kang, 2021). The prevalence of this group ranged from 8 to 70%; however, once readjusted, the prevalence was 20.10%, thus making it the second largest depressive symptom trajectory group (2446 individuals) (Table 2).

Multiple predictors were associated with the *increasing* depressive symptom trajectory group (Table 3). Fewer years of education were associated with membership to this group (Heath et al., 2023; Shen & Wang, 2023). Compared to the *stable low* depression group, individuals in the *increasing* depressive group were younger (Gomez et al., 2017; Heath et al., 2023), spent fewer days in hospital (Gomez et al., 2017), and were more likely to use antidepressants and/or counselling services post ABI (Bombardier et al., 2016). Additionally, one study found that individuals from ethnic minority groups were associated with membership to this group (Shen & Wang, 2023).

However, there were discrepancies in predictors between studies. For example, Heath et al. (2023) found that men were more likely to belong to the *increasing* depressive symptom group, whereas Shen and Wang (2023) found that being of male sex was associated with a decreased likelihood of belonging to this group. Furthermore, two studies identified that more severe TBI was associated with membership to the *increasing* depression group (Bombardier et al., 2016; Martinez et al., 2024); in contrast, another study demonstrated that having a less severe brain injury was associated with being in this group (Gomez et al., 2017).

## Discussion

This systematic review investigated longitudinal patterns of depressive symptoms following an ABI by identifying and characterising distinct groups of individuals who follow various trajectories of depressive symptoms. Our review of ten studies identified four common depressive symptom trajectories: *stable low*, *persistent high*, *decreasing*, and *increasing* depressive symptoms. Additionally, the review examined predictors of group membership which varied between groups.

Our findings indicate that the majority of individuals (68%) belonged to the *stable low* depressive symptom trajectory group after a brain injury, suggesting that a substantial proportion of individuals maintains low levels of depressive symptoms following such an event. This is consistent with the existing brain injury literature, which has shown many individuals do not exhibit mood disturbances post-injury (Gould et al., 2011a, b; Jorge et al., 2004).

Our review revealed that nearly 11% of individuals experience an improvement in their depressive symptoms over time. One study revealed that less severe injury severity was a significant predictor of membership in the *decreasing* depressive symptom group (Martinez et al., 2024). Individuals with less severe brain injuries may experience fewer impairments in their cognition and daily functioning, which may result in a reduced disruption to their lifestyle (Draper & Ponsford, 2008). Such individuals are also likely to undergo a faster physical recovery, alleviating the burden of long-term disability or pain. These factors combine to reduce the levels of physical and psychological stress over time. As a result, the quicker restoration of normal routines and roles in personal and professional domains may foster emotional resilience, possibly contributing to a more rapid reduction in depressive symptoms (Hanks et al., 2016; Wardlaw et al., 2018). Few predictors were identified for the *decreasing* depressive symptom trajectory group, likely due to the limited scope of predictors assessed across the five studies. These studies may have overlooked various influences that could contribute to recovery. Future research should explore whether factors such as treatment modalities, therapeutic interventions, and social support act as predictors for this group, considering that prior research has demonstrated significant associations between these elements and reduced depression following brain injury (Agtarap et al., 2018; Juengst et al., 2017).

While a significant proportion of individuals recover well after a brain injury, our review revealed that over one-third of individuals either develop or continue to experience persistent depressive symptoms. These findings expand our understanding that the effects of ABI extend far beyond the initial acute recovery period. Our review indicated that 13% of individuals experienced *persistent high* depressive symptoms over time. Although this figure is relatively low compared to the other groups, the prevalence of *persistent high* depressive symptoms following a brain injury is still more than double compared to the rates of chronic depression in the general adult population, which is an estimated five percent (Murphy & Byrne, 2012; Rubio et al., 2011).

Additionally, a consistent finding across the reviewed studies was that a group of individuals with initially low levels of depression exhibited an increase in depressive symptoms over time following a brain injury. This group accounted for the second largest prevalence (20%), underscoring the need for long-term monitoring of depressive symptoms. However, most studies have focused on the impact of depression within the first year following a brain injury, often neglecting the long-term prevalence. This review highlights that increasing depression is a prevalent occurrence in studies assessing depression beyond the one-year mark, extending up to a decade (Shen & Wang, 2023) and even 30 years after the injury (Kumar et al., 2021). Moreover, increased awareness and emphasis are needed on persistent and worsening depression after a brain injury given its cascading adverse effects on quality of life (QoL). Studies that have examined the trajectories of QoL following a brain injury using group-based trajectory modelling have found similar patterns including high, low stable, improving, and worsening QoL trajectories (Juengst et al., 2015; Pucciarelli et al., 2019; van Mierlo et al., 2018; Wiechman et al., 2023). Interestingly, Pucciarelli et al. (2019) found that these QoL trajectories mirrored changes in depression patterns, revealing a negatively correlated relationship between the two: when depression increases, QoL decreases concurrently.

The review also identified a range of predictors for group membership to the *persistent high* and *increasing* depressive symptom trajectory groups. The *persistent high* and *increasing* groups had some similar predictors such as higher injury severity (Bombardier et al., 2016; Gomez et al., 2017; Ren et al., 2017). More severe brain injuries typically cause greater damage, leading to more significant cognitive and physical deficits compared to milder injuries (de Guise et al., 2017). These more pronounced cognitive and physical deficits may impede their daily functioning and ability to participate in their community, which can consequently lead to decreased QoL and higher depression rates (Pucciarelli et al., 2019). Additionally, other predictors identified for both groups included fewer days in hospital (Gomez et al., 2017) and association with both sexes (Gomez et al., 2017; Heath et al., 2023; Kumar et al., 2021). These predictors emphasise the importance of monitoring key factors in primary care settings to ensure that individuals at risk of developing or experiencing persistent depressive symptoms receive the best care and ongoing monitoring available.

Additionally, younger age (Gomez et al., 2017; Heath et al., 2023) and fewer years of education (Heath et al., 2023; Shen & Wang, 2023) were common predictors of the *increasing* depressive symptom group. Heath et al. (2023) identified the mean age of their *increasing* depressive symptom group as 27.40 years old, whereas Gomez et al. (2017) did not provide the average age for this group; however, the mean age of their overall sample was 35.96 years old. It is possible that individuals within this age group may be more susceptible to *increasing* depressive symptoms due to post-injury difficulties with handling normal life stressors including their occupation and family responsibilities (Ownsworth et al., 2011). When faced with failures in their ability to resume their pre-injury roles, they may be forced to confront the idea that their impairments are long-term issues, which may trigger a reaction such as symptoms of depression (Roy et al., 2018). Moreover, individuals with fewer years of education may have less access to resources that could help mitigate stress such as stable employment or healthcare. They also may have lower health literacy, making it more difficult for them to recognise symptoms of depression early (National Academies of Sciences, 2022).

The review revealed inconsistent findings when exploring predictors of group trajectory membership. Discrepancies were found in sex in both the *persistent high* and *increasing* depressive symptom group, where Gomez et al. (2017) and Shen and Wang (2023) respectively concluded that females were more likely to belong to these depressive symptom groups, yet Kumar et al. (2021) and Heath et al. (2023) found the same findings for male participants. The inconsistent findings between studies could be due to the varied sex distribution within the subgroups. However, all studies do not provide descriptive statistics for each group and the original data which is required to investigate this idea. Moreover, inconsistent results were revealed for injury severity in the *increasing* depressive symptom group. Variations in how injury severity was measured across TBI studies may have resulted in differing conclusions. For instance, Gomez et al. (2017) utilised the length of post-traumatic amnesia and found that less severe injury severity was associated with membership in the *increasing* depressive symptom group. In contrast, Bombardier et al. (2016) and Martinez et al. (2024) employed the Glasgow Coma Scale and found that more severe injury severity was linked to membership in the same group. The different results generated by these scales can be attributed to their varying sensitivity and focus. The duration of post-traumatic amnesia may provide a more direct measure of cognitive recovery and long-term functional outcomes (Walker et al., 2018). On the other hand, the Glasgow Coma Scale assesses consciousness levels immediately following injury, which might not capture subtler or long-term cognitive deficits as effectively. Studies comparing these assessment tools suggest that while the Glasgow Coma Scale is useful for initial triage and prognosis, the length of post-traumatic amnesia may offer more accurate predictions of long-term outcomes (Konigs et al., 2018; Perrin et al., 2015). Consequently, the clinical presentation used to measure injury severity can significantly influence study results, underlining the importance of standardising injury severity measurements to ensure consistency and comparability across research studies.

### The Potential Moderating Effect of Brain Injury Type

A key consideration for this review is the potential moderating effect of brain injury type. Given the limited number of studies in this area, this review adopted a broad approach by examining ABI as an umbrella term encompassing both TBI and stroke, rather than restricting the review to a single aetiology. While post-stroke depression is recognised as having distinct clinical and neurobiological underpinnings (Robinson & Jorge, 2016), the findings of this review suggest that, despite some differences, TBI and stroke studies reveal similar patterns of depressive symptom trajectories. Notably, the *increasing* depressive symptom trajectory was identified across all studies, regardless of brain injury type, underscoring the shared risk of worsening depressive symptoms over time following ABI. Additionally, most studies reported a *stable low* depressive symptom trajectory, with the exception of one TBI and one stroke study. Furthermore, 57% of TBI studies and 67% of stroke studies identified a *persistent high* depressive symptom trajectory. The consistency in depressive symptom trajectories across both injury types suggests that, despite differences in neurobiological mechanisms and injury characteristics, a substantial proportion of individuals with TBI and stroke follow comparable long-term patterns of depressive symptoms.

In this review, the potential moderating effects of brain injury type are most evident in the *decreasing* depressive symptom trajectory group. This group was identified in 43% of TBI studies and 67% of stroke studies, suggesting a more pronounced recovery trajectory in the stroke population. This finding is interesting, as one might expect greater recovery trajectories in the TBI population, given their younger age and lower rates of secondary health conditions (Martinez et al., 2024). Several factors could explain this discrepancy. For instance, stroke survivors may experience better recovery due to higher levels of perceived social support (Elloker & Rhoda, 2018; Kruithof et al., 2013), whereas individuals with TBI report lower perceived social support and reduced social reintegration (Kersey et al., 2024; Zeng et al., 2016). Additionally, differences in recovery patterns between stroke and TBI populations may be influenced by methodological variations across studies, such as differences in sampling strategies and assessment timepoints. However, due to the limited number of studies included in this review, it remains difficult to determine whether these differences reflect true variations in depressive symptom trajectories between stroke and TBI, or whether they are artefacts of study design and methodological inconsistencies. Thus, the results should be interpreted with caution and underscore the need for further research to clarify whether specific depressive trajectories are inherently characteristic of one injury type, or if these variations arise due to differences in study design and participant sampling.

## Clinical Implications

This review highlights the variability and complexity of depressive symptoms following a brain injury and provides a deeper understanding of the natural course of depressive symptoms after such injury. The findings emphasise the importance of monitoring and screening for mood disturbance repeatedly, given that a large group of individuals develop depressive symptoms post-injury. Methods such as self-report depressive measures completed by the individual or an informant can be used which are straightforward and easy to administer. Regular use of depression self-reports can help healthcare providers detect early signs of depression and intervene promptly, potentially mitigating the severity of symptoms and improving overall outcomes.

From a clinical perspective, identifying predictors for depressive symptom trajectories after a brain injury is vital in recognising individuals who are at higher risk of persistent, or worsening depressive symptoms. By pinpointing factors that contribute to these trajectories, clinicians can better anticipate which individuals are likely to experience chronic or escalating depression. This allows for the implementation of tailored, proactive interventions that take into account the individual’s unique history, vulnerabilities, and risk factors. Personalised care strategies developed in this way can more effectively address the specific needs of each individual, possibly leading to improved mental health outcomes (Savulich et al., 2018). Furthermore, identifying at-risk individuals early, particularly in an acute care setting, enables timely education for both the individual and their family about the possible progression of depressive symptoms. Providing this information early on can reduce uncertainty, facilitate better planning, and empower both the individual and their support network to prepare for challenges commonly associated with brain injury recovery.

## Limitations

While this review provides insight into the long-term depressive symptom trajectories in ABI, it is subject to limitations. The reviewed studies exhibited substantial variability in methodology and sample composition, which poses challenges for direct comparison and synthesis of results. Differences in sample size, assessment tools, injury severity, type of injury, time post-injury, and inclusion/exclusion criteria were noted across the studies. In particular, assessment time points for assessing depression severity varied from a few months to 30 years post-injury, which complicates comparisons and potentially accounts for inconsistencies in the findings. These methodological discrepancies highlight the need for standardised approaches in future research to enhance the comparability and reliability of results.

A potential limitation of this review is the age-related differences between TBI and stroke populations, which may introduce an age confound in the interpretation of depressive symptom trajectories. The stroke studies generally included older participants, whereas TBI studies primarily involved younger and middle-aged adults. The higher risk of post-stroke depression in older adults may partly reflect age-related vulnerabilities such as cognitive decline, chronic illness, and social isolation rather than injury-specific effects (Robinson & Jorge, 2016). Conversely, younger individuals with TBI may experience different psychosocial stressors, such as difficulties returning to work or education, which could influence their depressive symptom trajectory (Rapoport, 2012). However, our finding showed that both injury types exhibited similar patterns for the *stable low*, *persistent high*, and *increasing* depressive symptom trajectory groups. This suggests that factors beyond age may be more influential in shaping long-term depressive outcomes. Nonetheless, the potential confounding effect of age cannot be overlooked; hence, future research should examine age as a potential moderator to better disentangle the effects of age from injury type in shaping long-term depressive outcomes.

Additionally, our review was heavily reliant on self-reported measures of depressive symptoms. While self-report scales are valid and widely used in brain injury research, they can be influenced by the deficits in awareness, which is common among individuals with ABI (Simmond & Fleming, 2003) and can affect the accuracy of the depressive symptom scores. Moreover, in seven studies, the self-reports were completed by the participant, whereas in the other three, they were completed by the researcher via an interview. It is likely that there will be discrepancies between depressive scores due to this methodological difference (Satz et al., 1998). Consequently, the use of diverse depressive measures and methods of administering these measures across the reviewed studies further complicates the synthesis of findings. Incorporating informant ratings in future research could provide a more comprehensive and nuanced understanding of depressive symptoms.

All predictors were identified exclusively from TBI studies, which limits the generalisability of our findings to other types of brain injuries, such as stroke and other forms of ABI. Future stroke and other ABI research would benefit from more comprehensive analyses of predictors influencing depressive trajectory group membership. Furthermore, the existing studies have not examined a broad range of predictors, despite the complexity and variability of depression following a brain injury. Given the diverse factors that influence post-injury depression, future research should incorporate a wider spectrum of potential predictors. Key contributors such as sleep disturbances, fatigue (Ponsford et al., 2012), chronic pain (Khoury & Benavides, 2018), and comorbid conditions (Stein et al., 2019) have been shown to significantly impact depressive symptoms and should be considered in future analyses. Including these variables is essential for capturing the multifaceted nature of depression in the context of brain injury.

Moreover, understanding early predictors of post-injury recovery is critical for guiding early intervention strategies (Veerbeek et al., 2011). Previous research has explored early predictors of post-injury depression such as injury severity (Rabi-Zikic et al., 2020), disability (Ayerbe et al., 2013), and age at injury (Levin et al., 2005). Yet, this review indicates that early predictors of different depressive symptom trajectories remain underexamined. Identifying these predictors is essential for distinguishing between individuals who may recover well post-injury and those who require longer-term support. Future research should focus on early post-injury indicators, such as acute stress responses and early mood disturbances, as potential risk factors for long-term depression trajectories. This knowledge can then inform the development of timely interventions and targeted treatment strategies, ultimately improving long-term outcomes for individuals with brain injuries.

## Conclusion

Overall, this systematic review elucidates the complex and varied nature of depressive symptom trajectories following an ABI. The identification of four primary depressive symptom trajectories that appear most frequently in studies—*stable low*, *persistent high*, *decreasing*, and *increasing*—underscores the heterogeneity of depressive experiences post-brain injury. Although the majority of people maintain low depressive symptoms, a significant portion experience persistent or increasing depression, necessitating long-term monitoring. This review also highlighted a range of predictors associated with different trajectories, which is vital for creating personalised interventions for affected individuals. Future research should standardise methodologies and expand to diverse brain injury types to enhance the generalisability and applicability of these findings.

The author, PP, was awarded the Australian Government Research Training Program Stipend.

## Supplementary Information

Below is the link to the electronic supplementary material.

Supplementary file1 (DOCX 38.1 KB)

Supplementary file2 (DOCX 31.8 KB)
