# Supplementary material for: Characterising Long-Term Depressive Symptoms Post-brain Injury: A Systematic Review of Symptom Trajectory Groups and Their Predictors
Source: Neuropsychol Rev. 2025 Aug 5;36(3):321–38. doi: 10.1007/s11065-025-09674-6 (PMC13653874; doi:10.1007/s11065-025-09674-6)
Supplement: Supplementary file 1 [file 11065_2025_9674_MOESM1_ESM.docx]

**Table S1**

**Search strategy for all databases**

|  | **Concept 1** | **AND** | **Concept 2** | **AND** | **Concept 3** |
| --- | --- | --- | --- | --- | --- |
| **Medline** | (exp Brain Injuries/ OR exp Brain Injuries, Traumatic/) OR (head OR brain OR crani* OR cerebr*) ADJ2 (injur* OR incident* OR trauma* OR damage* OR accident*) OR (brain-ischemi*) OR stroke* OR (diffuse-axonal-injur*) OR TBI OR ABD OR ABI |  | (exp Depression/ OR exp Psychological Distress/) OR depress* OR MDD OR (major-depressive-disorder) OR (depressive-disorder) OR (major-depression) OR (psychological-distress) |  | (group-based-trajectory-model*) OR (growth-mixture-model*) OR (latent-growth-curve-model*) OR (latent-class-growth-analys*) |
| **ScienceDirect** | (stroke OR (“brain injury”) OR (“acquired brain injury”) OR (“traumatic brain injury”) |  | (depression OR ("psychological distress") |  | ("group based trajectory model") OR ("growth mixture model") OR ("latent growth curve analysis”) |
| **Scopus** | ("brain ischemia" ) OR stroke OR ("brain injury") OR ("acquired brain injury") OR ("traumatic brain injury") OR ("diffuse axonal injury") OR tbi OR abd OR abi |  | depression OR mdd OR ( "major depressive disorder" ) OR ( "major depression" ) OR ( "psychological distress" =) |  | ("group based trajectory model") OR ("growth mixture model") OR ("latent growth curve model") OR ("latent class growth analysis") |
| **CINHAL plus** | (MH "Brain Injuries+") OR (MH "Stroke+") OR (head OR brain OR crani* OR cerebr*) ADJ2 (injur* OR incident* OR trauma* OR damage* OR accident*) OR (brain-ischemi*) OR stroke* OR (diffuse-axonal-injur*) OR TBI OR ABD OR ABI |  | **(**MH "Depression+") OR (MH "Psychological Distress+") OR depress* OR MDD OR (major-depressive-disorder) OR (depressive-disorder) OR (major-depression) OR (psychological-distress) |  | (group-based-trajectory-model*) OR (growth-mixture-model*) OR (latent-growth-curve-model*) OR (latent-class-growth-analys*) |
| **Web of Science** | (head OR brain OR crani OR cerebr) ADJ2 (injur OR incident OR trauma OR damage OR accident) OR ("brain-ischemia") OR stroke OR ( "brain-injury" ) OR ( "acquired-brain-injury" ) OR ( "traumatic-brain-injury" ) OR ("diffuse-axonal-injury") OR TBI OR ABD OR ABI |  | depression OR MDD OR (“major-depressive-disorder”) OR (“depressive-disorder”) OR (“major-depression”) OR (“psychological-distress”) |  | ("group-based-trajectory-model") OR ("growth-mixture-model") OR ("latent-growth-curve-model") OR ("latent-class-growth-analysis") |
| **PubMed** | ("Brain Injuries"[Mesh] OR "Brain Injury, Chronic"[Mesh] OR "Brain Injuries, Traumatic"[Mesh] OR "Brain Injuries, Diffuse"[Mesh] )) AND ( "Stroke"[Mesh] OR "Ischemic Stroke"[Mesh] OR (head OR brain OR crani* OR cerebr*) ADJ2 (injur* OR incident* OR trauma* OR damage* OR accident*) OR (brain-ischemi*) OR stroke* OR (diffuse-axonal-injur*) OR TBI OR ABD OR ABI |  | ("Depression"[Mesh] AND "Depressive Disorder"[Mesh]) AND "Psychological Distress"[Mesh] OR depress* OR MDD OR (major-depressive-disorder) OR (depressive-disorder) OR (major-depression) OR (psychological-distress) |  | (group-based-trajectory-model*) OR (growth-mixture-model*) OR (latent-growth-curve-model*) OR (latent-class-growth-analys*) |

*Note.* Date of last search for all databases were 6^th^ of September 2024. Filters included for all databases were: ‘English language’ and ‘full text’.

**Table S2**

Questions used from Joanna Brigg’s critical appraisal tool for prevalence studies to evaluate the methodological quality of each study (Munn et al., 2020)

| **Questions** | |
| --- | --- |
| Q. 1 | Was the sample frame appropriate to address the target population? |
| Q. 2 | Were study participants recruited in an appropriate way? |
| Q. 3 | Was the sample size adequate? |
| Q. 4 | Were the study subjects and setting described in detail? |
| Q. 5 | Was data analysis conducted with sufficient coverage of the identified sample? |
| Q. 6 | Were valid methods used for the identification of the condition? |
| Q. 7 | Was the condition measured in a standard, reliable way for all participants? |
| Q. 8 | Was there appropriate statistical analysis? |
| Q. 9 | Was the response rate adequate, and if not, was the low response rate managed appropriately? |

Munn, Z., Moola, S., Lisy, K., Riitano, D., & Tufanaru, C. (2020). Systematic reviews of prevalence and incidence. In JBI (Ed.), *JBI Manual for Evidence Synthesis*.

**Table S3**

Final consensus on methodological quality of each study

| *Study* | *Overall Risk* | *Q.1* | *Q. 2* | *Q. 3* | *Q.4* | *Q.5* | *Q.6* | *Q.7* | *Q. 8* | *Q. 9* |
| --- | --- | --- | --- | --- | --- | --- | --- | --- | --- | --- |
| Ayis et al., (2019) | Low | Yes | Yes | Yes | Yes | Yes | No | Yes | Yes | Yes |
| Kang, 2021 | Low | Yes | Yes | Yes | Yes | Yes | No | Yes | Yes | Yes |
| *Malhotra et al., (2016)* | Low | Yes | Yes | No | Yes | Yes | No | Yes | Yes | Yes |
| *Bombardier et al., (2016)* | Low | Yes | Yes | Yes | Yes | Yes | No | Yes | Yes | Yes |
| *Heath et al., (2023)* | Moderate | Yes | Yes | No | Yes | No | No | Yes | Yes | Unclear |
| *Gomez et al., (2017)* | Low | Yes | Yes | Yes | Yes | Yes | No | Yes | Yes | Yes |
| *Kumar et al., (2021)* | Low | Yes | Yes | Yes | Yes | Yes | No | Yes | Yes | Yes |
| *Martinez et al., (2024)* | Low | Yes | Yes | Yes | Yes | Yes | Yes | Yes | Yes | Unclear |
| *Ren et al., (2017)* | High | No | Unclear | No | Yes | No | Yes | Unclear | Yes | Unclear |
| Shen, 2023 | Low | Yes | Yes | Yes | Yes | Yes | No | Yes | Yes | Yes |

*Note. Risk of bias was assessed based on the proportion of ‘Yes’ responses to each item. Low risk of bias was determined if the studies had 70% or more ‘Yes’ responses, moderate risk was 50% to 69% ‘Yes’ responses and high risk of bias was determined for studies with 49% or fewer ‘Yes’ responses (Polmann et al., 2019). One study was deemed as high risk due to insufficient information provided to answer the quality assessment questions.*

**Table S4**

Available brain injury characteristics from the reviewed studies.

|  | *Martinez et al., (2024)* | *Bombardier et al., (2016)* | *Heath et al., (2023)* | *Gomez et al., (2017)* | *Kumar et al., (2021)* | *Ren et al., (2017)* | Shen & Wang (2023) | Ayis et al., (2019) | Kang (2021) | *Malhotra et al., (2016)* |
| --- | --- | --- | --- | --- | --- | --- | --- | --- | --- | --- |
| *n* | 1943 | 559 | 148 | 1074 | 3541 | 129 | 1989 | 2313 | 766 | 172 |
| *Injury type* | TBI | TBI | TBI | TBI | TBI | TBI | TBI | Stroke | Stroke | Stroke |
| *Average Age (SD)* | 39.72 (16.65) | 42.5 (17.9) | 38.7 (16.36) | 35.96 (16.61) | 73.35 (5.75) | 33.3 (14.60) | 18.79 (1.58) | 66.98 (14.45) | 77.35 (6.54) | 71.6 (12.2) |
| Cause of Injury | | | | | | | | | | |
| *Motor Vehicle Accident* | 1158 (49.5%) | 262 (46.9%) | 88 (60%) | 417 (39.2%) | - | - | - | - | - | - |
| *Fall* | 529 (22.6%) | 184 (32.9%) | 46 (32%) | 213 (20%) | - | - | - | - | - | - |
| *Assault/Violence* | 141(6.0%) | 63 (11.3%) | 8 (6%) | 291 (27.3) | - | - | - | - | - | - |
| *Sports related* | - | - | 4 (3%) | 75 (7%) | - | - | - | - | - | - |
| *Other/unknown* | 509 (21.7) | 50 (8.9%) | - | 68 (6.4%) | - | - | - | - | - | - |
| *TBI History (yes/no)* | 394(19.4%) | - | - |  | - | - | - | - | - | - |
| Average Admission GCS score | | | | | | | | | | |
| *Mild group* | 14.8 | - | - | - | - | - | - | - | - | - |
| *Mod – severe group* | 6.3 | - | - | - | - | - | - | - | - | - |
| GCS | | | | | | | | | | |
| *Highly severe (3-4)* | - | 140 (25%) | - | 17 (1.7%) | - | 18 (14%) | 404 (20.50%) | - | - | - |
| *Moderate severe (5-8)* | - |  | - |  |  | 111 (86%) |  | - | - | - |
| *Moderate (9-12)* | - | 128 (22.9%) | - | 276 (26.6%) | - | - | 165 (8.37%) | - | - | - |
| *Mild (13-15)* | - | 291 (52.1%) | - | 743 (35.85%) | - | - | 269 (13.65%) | - | - | - |
| GCS (verbal) | | | | | | | | | | |
| *Not fully oriented* | - | - | - | - | - | - | - | 13.8% | - | - |
| *Fully oriented* | - | - | - | - | - | - | - | 42.97% | - | - |
| *Unknown* | - | - | - | - | - | - | - | 43.23% | - | - |
| *Avg PTA (hours)* | - | - | - | 2.26 | - | - | - |  | - | - |
| Length of PTA | | | | | | | | | | |
| *< 5 minutes* | - | - | 1(1%) | - | - | - | - | - | - | - |
| *1-24 hours* | - | - | 1 (1%) | - | - | - | - | - | - | - |
| *1-7 days* | - | - | 31 (21%) | - | - | - | - | - | - | - |
| *1-4 weeks* | - | - | 83 (56%) | - | - | - | - | - | - | - |
| *> 4 weeks* | - | - | 31 (21%) | - | - | - | - | - | - | - |
| During of LOC | | | | | | | | | | |
| *Less than 1 hour* | - | - | - | - | 355 (70.7%) | - | - | - | - | - |
| *1 hour or more* | - | - | - | - | 73 (14.6%) | - | - | - | - | - |
| *Unknown* | - | - | - | - | 72 (14.7%) | - | - | - | - | - |
| *Cerebral Contusion (yes)* | - | 162 (29%) | - | - | - | - | - | - | - | - |
| *Intracranial Haemorrhage* | - | 384 (68.7%) | - | - | - | - | - | - | - | - |
| *Avg Age at injury* | - | - | 38.70 | - | - | - | - | - | - | - |
| *No. of Hosp days after injury* | - | - | - | 5.70 | - | - | - | - | - | - |
